# Modern Aspects of Appendicitis.—I

**Published:** 1893-11-25

**Authors:** 


### Modern Aspects of Appendicitis.—I.

This comparatively new word marks a distinct era in our knowledge of a most important disease. It is a strange fact that our knowledge of the appendix vermiformis has always been behindhand. Its existence was not even recognised till 1524, and it was long after this before it was associated with pathology. It is only quite recently that we have gained any accurate facts as to its anatomy, and for information on this point we are much indebted to Professor Struthers, who, in the October number of the *Edinburgh Medical Journal*, points out that the appendix is very variable in place, mode of origin, direction, and length. It usually arises about half way up the back of the cœcum generally to the inside of the middle. There is no valve at the entrance to it, but only a sharp crescentic edge, which does not offer any impediment to egress or ingress. Its direction is first upwards to about the level of the top of the cœcum proper, for which part of its course it is bound to the cœcum by peritoneal covering at the back of this, then downwards and inwards, showing itself below the ileum pendulous and free with a narrow triangular mesentery. Its length varies from three-quarters of an inch to six inches, and even a length of nine inches has been recorded. Its average diameter is a quarter of an inch. It is most important for the clinical observer to bear in mind that all these facts are liable to numerous variations; indeed, probably there is no organ in the human body which has wider deviations from the normal than the vermiform appendix. This is not surprising when we remember that it is a vestigial structure representing an organ of great use in the lower animals, but only a nuisance in man, for in him it has no function, but is often the seat of serious disease, making us wish that we had arrived at the period at which it would have quite disappeared.

Until lately it was assumed that many, if not all, the cases of inflammation around the cœcum and appendix arose in the loose cellular tissue behind the cœcum, and hence the common use of the term perityphlitis, but gradually clinical observers have come round to the opinion that such is not the case, and that the trouble in these cases nearly always originates in the appendix. This is quite borne out by Professor Struthers, for he shows that the anatomy books are in error in stating that the peritoneum does not completely surround the cœcum, for it entirely covers it and forms a mesentery for it, and consequently there is no loose areolar tissue behind it. It is curious that this erroneous teaching should die so hard, and that the words perityphlitis, typhlitis, paratyphlitis, iliac phlegmon, &c., should not, till now, have begun to give way to the new term appendicitis, for Parkinson, as long ago as 1812, recorded a case of peritonitis due to perforation of the appendix, and many other observers, as Wilks, in 1855, placed cases on record.

Before leaving the anatomy of the subject, we may point out that as the appendix is free, and often of considerable length, it may form adhesions to any surrounding structures; thus it has been known to adhere to the ovary omentum, rectum bladder, abdominal wall, &c. This adhesion may greatly increase the clinical and operative difficulties of a case.

There will in future be no excuse for ignorance on the subject of appendicitis, or for the confusion of it with typhlitis, perityphlitis, &c., for Dr. Kelynack has recently published a most exhaustive thesis on the whole subject, and we will now give some of the conclusions to which he has arrived.

Passing over his anatomical introduction, the first point of interest to the clinical observer is the account of the importance of hernia of the appendix. This usually takes place either into the sac of a right inguinal hernia, or into one of the very numerous pericœcal peritoneal fossæ.

The appendix is also dangerous from the fact that by its adhesions to surrounding organs it forms a band under which other parts of the gut may slip and become strangulated. With regard to the foreign bodies found in it, Kelynack quite confirms the general belief that genuine foreign bodies are very rare, and that hardened masses of fæces are very common. He mentions some interesting cases of calculi in the tip of the appendix.

As we have already indicated, we agree with him that typhlitis and perityphlitis are loose terms, and ought to be abolished, or at best only used in a vague way to mean any inflammation in the neighbourhood of the cœcum or appendix. Appendicitis is an exact term, and if the inflammation originating in the appendix spreads to the neighbouring parts, the phrase periappendicitis might be allowed. Cœcitis and pericœcitis would then be available to designate inflammation of and around the cœcum. The proportion of cases of cœcitis to appendicitis is probably only one to a hundred. Appendicitis itself may be subdivided into simple appendicitis, perforative appendicitis, recurrent appendicitis, tubercular appendicitis, typhoid appendicitis, and actinomycotic appendicitis.

Of these simple, appendicitis can be quickly dismissed, for very little is known about it. Frequently it is spoken of as catarrhl appendicitis, but this is a bad term, for it implies a knowledge we do not possess. Judging by post-mortem experience, signs of old simple appendicitis are found in about 8 per cent. of the bodies examined, although some observers have put the percentage higher. In our next article we shall deal with the other forms of appendicitis.

